# Two-dimensional transthoracic echocardiography versus two-dimensional trans esophageal echocardiography for assessment of moderate to large atrial septal defects and the device size in pediatric patients before transcatheter closure

**DOI:** 10.1186/s12887-026-06695-9

**Published:** 2026-04-01

**Authors:** Hani M. Adel, Ahmed El-Nawawy, Aly Abdel Mohsen, Omar Raafat

**Affiliations:** Department of Pediatrics, Faculty of Medicine, Alexandria University, Alexandria, Egypt

**Keywords:** Atrial septal defect, Transthoracic, Transesophageal, Transcatheter, Echocardiography

## Abstract

**Background:**

Atrial Septal Defects (ASDs) are a common type of congenital heart defect (CHD) in children. Accurate pre-procedural assessment is crucial for successful transcatheter closure. This study aims to compare ASD size and surrounding rims measurements from transthoracic echocardiography (TTE) and transesophageal echocardiography (TEE) in pediatric patients undergoing transcatheter closure of ASDs, assess agreement between TTE and TEE measurements, and evaluate TTE’s potential as a first-line imaging modality for ASD assessment.

**Methods:**

This prospective cohort study was conducted on 50 pediatric patients with secundum ASD admitted for transcatheter device closure of ASD in Smouha University Hospital. Patients underwent TTE and TEE examinations before undergoing the procedure. ASD size and rim measurements (aortic, AV valve, SVC, IVC, posterior) were compared between modalities. Agreement was assessed using Bland-Altman analysis.

**Results:**

TTE measurements yielded significantly larger values of ASD size compared to TEE (22.27 ± 8.64 mm vs. 14.93 ± 5.11 mm; *p* < 0.001). Significant differences were also observed in rim measurements for the AV valve, Aortic, Posterior, and IVC rims (*p* < 0.05), while SVC rim differences were not significant (*p* = 0.212). Despite mean differences, a strong positive correlation was discovered between TTE and TEE measurements of ASD size (rs = 0.963, *p* < 0.001). Bland-Altman analysis demonstrated good agreement with less than 5% of points falling outside the 95% limits of agreement. Procedural success was 100% with no residual shunts or complications at 24-hour follow-up.

**Conclusions:**

TTE was found to be reliable and cost-effective for evaluation of secundum ASDs, showing strong correlation with TEE. While TTE measurements were systematically larger, the agreement supports its use as a first-line modality for uncomplicated cases, while TEE remains valuable for detailed evaluation in complex scenarios.

## What is Known?

1. Transesophageal echocardiography (TEE) is the gold standard for accurate atrial septal defect (ASD) assessment before transcatheter closure.
2. Transthoracic echocardiography (TTE) is safer, cheaper, and non-invasive but may underestimate rims or defect size.

## What is New?

1. Our study shows strong correlation and agreement between TTE and TEE in pediatric ASD assessment despite systematic measurement differences.
2. TTE can reliably guide device sizing, supporting its use as a first-line modality, reserving TEE for complex or inconclusive cases.

## Introduction

Atrial septal defects (ASDs) represent one of the most frequently encountered congenital cardiac anomalies in children, with an estimated incidence of approximately 1 per 1,000 live births [1]. Advances in catheter-based interventions have established transcatheter device closure as the preferred treatment option for appropriately selected secundum ASDs, owing to its minimally invasive nature and favorable outcomes [2].

Successful transcatheter closure depends largely on precise pre-procedural assessment of defect size, morphology, and the adequacy of surrounding rims. Echocardiography remains the cornerstone imaging modality for this evaluation [3]. Two-dimensional transthoracic echocardiography (TTE) is widely available, cost-effective, and well tolerated in pediatric populations; however, its diagnostic accuracy may be influenced by patient anatomy and acoustic window limitations [4–6].

In contrast, transesophageal echocardiography (TEE) allows closer proximity to the atrial septum and typically provides higher spatial resolution, facilitating detailed visualization of ASD margins and adjacent structures (7,8). This advantage has led to TEE being commonly regarded as the reference technique for ASD assessment and intraprocedural guidance. Nevertheless, TEE is invasive, frequently requires general anesthesia in children, and is associated with increased procedural cost and patient discomfort [9].

The optimal imaging strategy for pediatric ASD assessment remains debated. While several studies have demonstrated acceptable agreement between TTE and TEE measurements, others have highlighted the superior delineation of rims and defect geometry provided by TEE, particularly in complex or large defects [3, 6]. Given the limited pediatric-specific comparative data and the clinical implications of imaging selection, further evaluation of the agreement between TTE and TEE in routine practice is warranted [7, 8, 10, 11].

Existing comparative studies in pediatric populations are limited, and consensus on the agreement between TTE and TEE for comprehensive ASD assessment in children is lacking. Therefore, this study was necessary to systematically compare TTE and TEE measurements, evaluate their correlation and agreement in a pediatric cohort, and assess the potential of TTE to serve as a reliable first-line imaging modality for ASD evaluation prior to device closure. Accordingly, this study was designed to systematically compare ASD size and rim measurements obtained by TTE and TEE in a pediatric cohort undergoing transcatheter closure, and to assess whether TTE can reliably serve as an initial imaging modality for procedural planning.

## Materials and methods

### Study design and population

This was a prospective cohort study conducted on 50 pediatric patients with secundum ASD admitted for transcatheter device closure of ASD in Smouha University Hospital. This study was conducted from May 2024 to July 2025.

Ethical approval for this study was granted by the Alexandria University Ethical Committee (date: 10.05.2022, Serial No. 0201787). Written informed consent was obtained from the parents or legal guardians of all participating children prior to enrollment, in accordance with the Declaration of Helsinki and its 2020 amendments.

The sample size was determined based on the number of eligible consecutive patients admitted during the study period who met the inclusion criteria; A post-hoc power analysis based on the observed correlation coefficient (rs = 0.963) demonstrated that a sample size of 50 patients provided > 90% power to detect significant correlation at α = 0.05.

The inclusion criteria comprised patients less than 18 years old with symptomatic or asymptomatic but hemodynamically significant ostium secundum ASDs amenable for device closure. Also, the distance to the defect is required to be at least 5 mm from the atrio-ventricular (AV) valve, right upper pulmonary vein, coronary sinus, inferior vena cava, superior vena cava, and posterior rim. The exclusion criteria included children with other congenital heart diseases, sinus venosus ASDs, primum type ASDs, coronary sinus defects, insufficient atrioventricular rim (less than 5 mm), or severe pulmonary hypertension.

### Echocardiographic assessment

All patients underwent both TTE and TEE examinations prior to the transcatheter closure procedure using the Philips EPIQ 7 echocardiography system, X5-1 probe, TEE probe, Andover, MA, USA. TEE examinations were performed by experienced pediatric cardiologists using dedicated TEE probes compatible with the echocardiography machines. Examiners were aware of the planned procedure but measurements were recorded independently prior to device selection.

### TTE and TEE image acquisition and measurements

Standardized protocols were followed for TTE and TEE image acquisition. Experienced cardiologists performed the examinations, ensuring optimal image quality for measurements. The larger diameters of the ASD were measured at different views of 2D TTE and 2D TEE and the device size was determined according to the largest measured ASD diameter [12]. Final device selection was primarily based on intra-procedural TEE measurements confirmed by fluoroscopy, though pre-procedural TTE measurements were used for initial planning. Balloon sizing was not routinely performed; sizing was based on echocardiographic stretched diameter estimates.

### Measurements of ASD rims

The width and length of the aortic rim (AR), AV valve rim, inferior vena cava rim (IVC rim), superior vena cava rim (SVC rim) and posterior rim at 45° in TEE, were measured using both TTE and TEE.

### Intervention phase

All patients received intravenous injections of antibiotics (ceftriaxone at 50 mg/kg) 30 min before the procedure. They also received intravenous heparin (100 mg/kg) after femoral sheath insertion to ensure proper anticoagulation (activated clotting time exceeding 200 s). The procedure was performed under general anesthesia. The device type was either Amplatzer septal occluder (ASO), Occlutech septal occluder, or Lifetech Cera ASD occluder. The device was deployed under fluoroscopy with left anterior oblique (LAO) cranial (30°) and TTE and TEE guidance.

Post-intervention care was done in the post-cath intermediate care unit at Smouha University Hospital, including an ECG, chest X-ray postero-anterior and lateral views to delineate the device, follow-up echocardiography 1 day after the procedure, and oral acetylsalicylic acid (5 mg/kg/day) for 6 months. Procedural success was defined as correct device placement with no significant residual shunt on color Doppler at 24 h.

### Statistical analysis

Data analysis was performed using statistical software (SPSS). Descriptive statistics were used to summarize demographic data (age, weight, gender distribution) and procedural details. Paired-samples t-tests were used to compare ASD size and surrounding rim measurements obtained from TTE and TEE. Bland-Altman plots were generated to assess the agreement between TTE and TEE measurements, including calculation of mean difference and 95% limits of agreement. Pearson or Spearman correlation coefficients were calculated as appropriate. A p-value of ≤ 0.05 was considered statistically significant.

## Results

As described in Table 1, a total of 50 pediatric patients (58% male, 42% female) underwent transcatheter ASD closure; their age fell between 2.2 and 15.5 years (mean: 6.42 ± 4.38 years), and their weight ranged from 10.5 to 48 kg (mean: 22.82 ± 8.74 kg). Regarding procedural characteristics, Amplatzer devices were used for closure in 38 patients (76%), followed by Lifetech devices (7 patients, 14%) and Occlutech devices (5 patients, 10%).

**Table 1:** Baseline characteristics and procedural data (*n* = 50)

Parameter	Value
Demographic data	
Sex (Male/Female)	29 (58.0%) / 21 (42.0%)
Age (years), Mean ± SD	6.42 ± 4.38 (Range: 2.2–15.5)
Weight (kg), Mean ± SD	22.82 ± 8.74 (Range: 10.5–48.0)
Device Data	
Device Type (Amplatzer/Lifetech/Occlutech)	38 (76%) / 7 (14%) / 5 (10%)
Device Size (mm), Mean ± SD	20.6 ± 5.64 (Range: 10.0–38.0)
Device/ASD size ratio, Mean ± SD	1.24 ± 0.32
Total septal length (mm), Mean ± SD	43.5 ± 10.2
Procedure Details	
Procedure time (min), Mean ± SD	30.07 ± 3.68
Fluoroscopy time (min), Mean ± SD	6.95 ± 1.65
Outcomes	
Procedural Success	50 (100%)
Complications	0 (0%)
Residual Shunt at 24 h	0 (0%)

*SD* Standard deviation, *ASD* Atrial septal defect

The device size ranged from 10 mm to 38 mm. Total septal length range was (24–60) and the mean was (43.5 ± 10.2). The mean device-to-ASD size ratio was 1.24 ± 0.32. The device-to-weight ratio ranged from 0.87 ± 0.32. ASD size / weight > 1.5 was observed in 4 cases. Also, weight was less than 15 kg in 12 cases. The procedure time ranged from 27.0 to 40.0 min. Fluoroscopy time ranged from 4.7 to 8.2 min.

Table 2 represents different techniques of ASD device deployment with the left upper pulmonary vein (LUPV) technique represents 48% of cases followed by left atrial deployment technique represents 24% of cases then right upper pulmonary vein (RUPV) technique represents 12% then balloon assisted technique and left atrial disc engagement disengagement technique each represents 8% of cases. There were no documented complications either major or minor complications. All procedures were completed successfully (100% procedural success). No device embolization, significant residual shunt, arrhythmia, erosion, or vascular complications were observed. follow-up echocardiography at 24 h, 1 month and 3 months after closure showed no residual flow in all patients. Table 3 summarizes vital data in the ASD Size Measurement, where a statistically significant difference (*p* < 0.001) was observed between the ASD size measured by TTE and TEE. TTE measurements yielded significantly larger values (Mean 22.27 ± 8.64 mm) compared to TEE (Mean 14.93 ± 5.11 mm). Additionally, It presents the measurements of ASD rims, as statistically significant differences were observed between TTE and TEE measurements for: AV valve rim (*p* < 0.001), Aortic rim (*p* = 0.001), posterior rim (*p* < 0.001) and IVC rim (*p* = 0.014). However, no statistically significant difference was found for the SVC rim (*p* = 0.212).

**Table 2:** Techniques of device deployment (*n* = 50)

Technique of device deployment	No	%
Left atrial deployment technique	12	24
LUPV technique	24	48
RUPV technique	6	12
Balloon assisted ASD closure	4	8
Left atrial disc engagement–disengagement technique (LADEDT)	4	8

*LADEDT* Left atrial disc engagement–disengagement technique, *LUPV* Left upper pulmonary vein, *RUPV* Right upper pulmonary vein

**Table 3:** Comparison between TTE and TEE according to different parameters (*n* = 50)

Parameter	TTE (Mean ± SD)	TEE (Mean ± SD)	*P*-value
ASD size (mm)	22.27 ± 8.64	14.93 ± 5.11	< 0.001*
AV valve rim (mm)	10.10 ± 1.0	10.38 ± 1.02	< 0.001*
Aortic rim (mm)	4.34 ± 1.79	4.55 ± 1.87	0.001*
SVC rim (mm)	6.53 ± 0.70	6.60 ± 0.72	0.212
IVC rim (mm)	7.77 ± 1.32	7.88 ± 1.34	0.014*
Posterior rim (mm)	7.22 ± 0.91	7.40 ± 0.87	< 0.001*

p: *p* value for comparing between TTE and TEE

*AR* Aortic rim, *ASD* Atrial septal defect, *AV* Atrio-ventricular, *IVC* Inferior vena cava, *SD* Standard deviation, *SVC *Superior vena cava, *TEE* Transesophageal echocardiography, *TTE* Transthoracic echocardiography

*: Statistically significant at *p* ≤ 0.05

Despite the statistical difference in means, a strong positive correlation existed between TTE and TEE measurements (rs = 0.963, *p* < 0.001), as explained in Table 4. Bland-Altman analysis demonstrated good agreement between TTE and TEE for ASD size assessment, with less than 5% of points falling outside the 95% limits of agreement. The mean difference between methods was systematic, with TTE consistently measuring larger diameters (mean difference: 7.34 mm; 95% limits of agreement: -2.1 to 16.8 mm).

**Table 4:** Correlation and Agreement Analysis (*n* = 50)

Comparison	Correlation Coefficient (rs)	*P*-value	Bland-Altman Mean Difference (95% LoA)
ASD size TTE vs. ASD size TEE	0.963	< 0.001*	7.34 mm (-2.1 to 16.8 mm)
ASD size TTE vs. Device size	0.948	< 0.001*	-
ASD size TEE vs. Device size	0.969	< 0.001*	-

*ASD* Atrial septal defect, *TTE* Trans-thoracic echocardiography, *TEE* Transesophageal echocardiography, *r*_s_ Spearman coefficient, *LoA* Limits of Agreement

*: Statistically significant at *p* ≤ 0.05

**Fig. 1:**
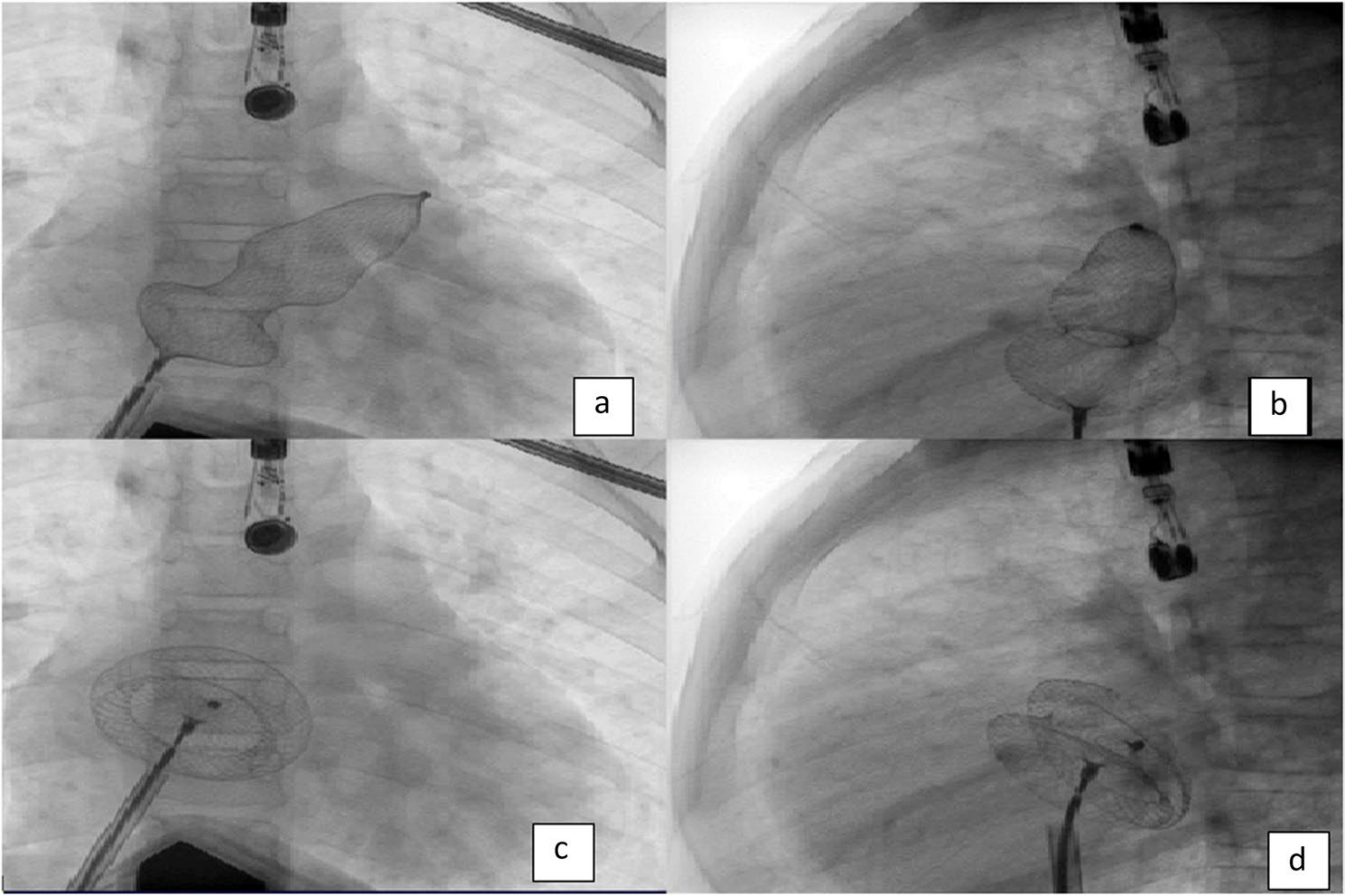
Fluoroscopy images demonstrating LADEDT via LUPV (**a**) represents left upper pulmonary vein engagement in AP view. (**b**) represents LUPV engagement on lateral view, (**c**) represents disengagement of the device to cover ASD with the device in place before device release in AP view. (**d**) shows the device before release in lateral view in place

**Fig. 2:**
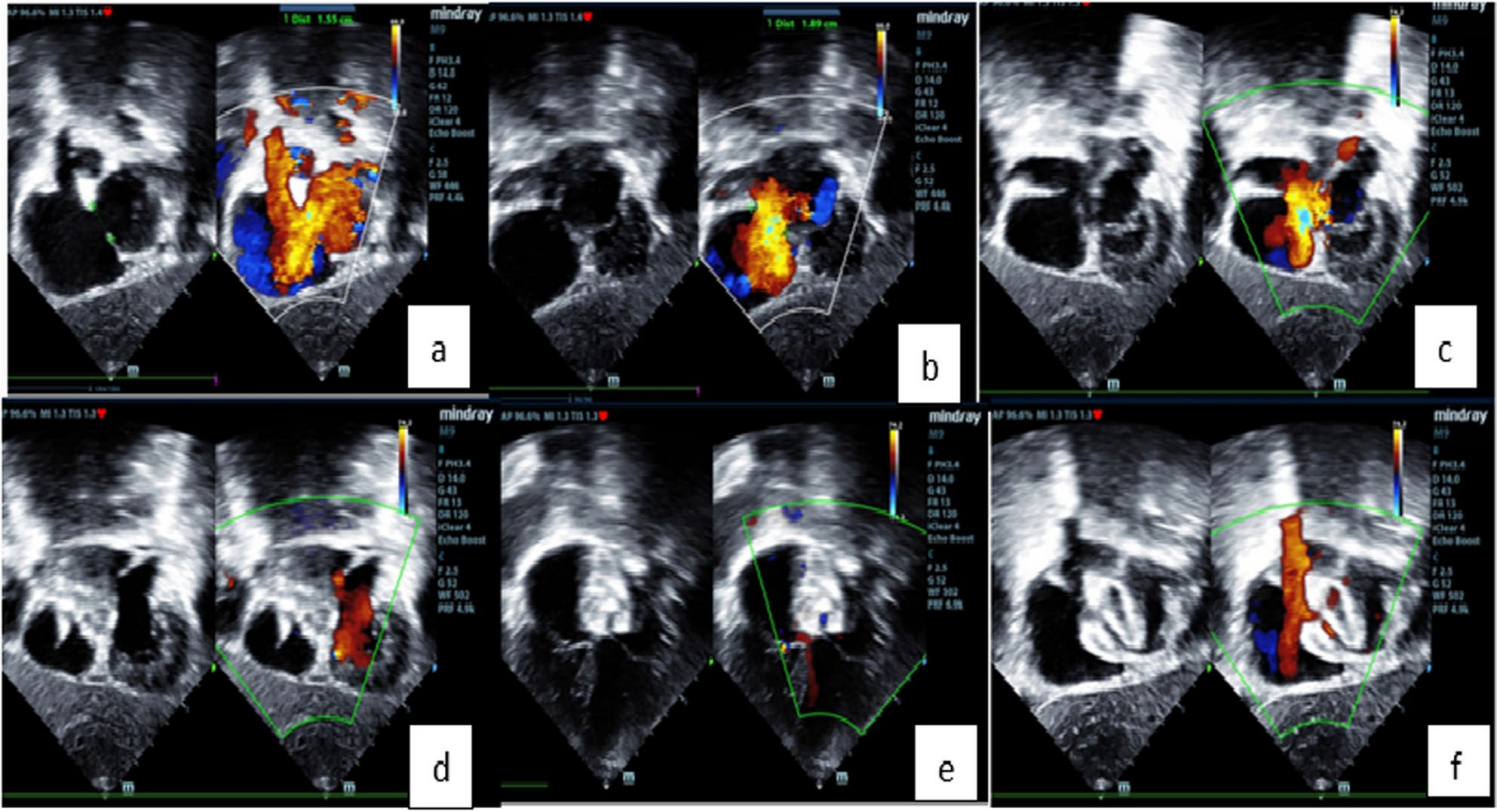
Transthoracic echocardiography–guided transcatheter ASD device closure using LADEDT. (**a**, **b**) shows ASD with left to right shunt in subcostal view using TTE with sheath inside LUPV. (**c**) shows introducing the device through the long sheath with left disc inside LUPV. (**d**) shows disengagement of the left disc with no flow detected by echo across ASD. (**e**, **f**) shows echo after device release in place with no residual flow

Table 4 and associated analysis showed that there was a positive correlation between ASD size by TTE and ASD size by TEE. Also, Bland-Altman plots affirmed a good agreement between ASD size measured by TTE or TEE and device size. There was a positive correlation between the ASD size using TTE and device size (rs = 0.948, *p* < 0.001). There was also a positive correlation between the ASD size using TEE and device size (rs = 0.969, *p* < 0.001) (Figs. 1 and 2).

## Discussion

The present study evaluated the agreement between transthoracic and transesophageal echocardiography for pre-procedural assessment of ASD size and device selection in pediatric patients. Our findings demonstrate that, although statistically significant differences exist between measurements obtained by the two modalities, TTE and TEE show strong correlation and clinically acceptable agreement regarding device selection outcomes.

In this study we used Amplatzer devices in the majority of cases (76%), followed by Lifetech (14%) and Occlutech (10%) devices. This aligns with other studies that show that Amplatzer devices are a widely accepted choice for ASD closure in children. For instance, Zakaria et al., who conducted a multicenter study in 9 French hospitals and used Amplatzer devices as the main occluders for ASDs [13]. Although Amplatzer devices predominated, outcomes were similar across device types; however, generalization to other devices should be cautious.

The mean device-to-ASD size ratio in this study is consistent with the recommendation that the device size should be 1–2 mm above the maximum stretched diameter of ASD for successful closure and minimizing complications [14, 15]. Mean procedure time and mean fluoroscopy time of this study are within the range of previously published studies on pediatric ASD closure [16].

In this cohort, TTE tended to yield significantly larger ASD measurements compared with TEE (22.27 mm vs. 14.93 mm). This finding contrasts with some literature where TTE may underestimate defect size due to acoustic window limitations [17, 18]. The observed overestimation by TTE in our cohort may reflect the influence of imaging planes and acoustic window variability inherent to transthoracic imaging, as well as differences in spatial resolution between modalities. TTE image quality can be affected by factors like body habitus and lung aeration, potentially leading to overestimation of the defect if the beam width artifact is not accounted for. Additionally, TEE provides a higher resolution image due to its closer proximity to the heart [19]. Although a statistically significant difference was observed between mean ASD diameter measured by TTE and TEE (22.27 ± 8.64 mm vs. 14.93 ± 5.11 mm), this discrepancy did not translate into adverse clinical outcomes or inappropriate device selection. Device size was selected based on the largest measured diameter obtained during comprehensive echocardiographic assessment, and all procedures were completed successfully without residual shunt, embolization, or rim-related complications. The strong correlation coefficient (rs = 0.963) and acceptable limits of agreement on Bland–Altman analysis support the clinical reliability of TTE measurements. Therefore, in uncomplicated secundum ASDs with adequate acoustic windows and well-visualized rims, TTE may provide sufficient information for device sizing and procedural planning, reserving TEE for complex anatomy or equivocal findings.

However, a strong positive correlation and good agreement between TTE and TEE measurements using the Bland-Altman plot were demonstrated. This finding is similar to those of other studies in children [20–24] in pediatrics, suggesting the potential of TTE for accurate size assessment, especially for uncomplicated secundum ASDs.

Rim measurements obtained by TEE were marginally larger for several anatomical margins, including the aortic and posterior rims. This finding is consistent with the enhanced visualization of septal anatomy afforded by esophageal imaging [25, 26]. Nonetheless, the observed differences did not translate into discrepancies in device selection or procedural success, as evidenced by the strong correlation between ASD size measured by TTE and the implanted device size.

We found positive correlation between the ASD size using TTE and device size (*p* < 0.001). Also, the Bland-Altman plot showed agreement between the ASD size using TTE and device size. Similar findings were reported by Vesal et al. [26]. Moreover, it was stated that both modalities can be used for device selection in experienced centers with successful outcomes [23, 24].

### Limitations

This study has several limitations. First, the relatively small sample size (*n* = 50) may limit the generalizability of the results to the broader pediatric ASD population and reduces statistical power. Second, the findings may not be representative of experiences in other institutions as it is a single-center study. Third, although the study was conducted prospectively, it was non-randomized and single-centered, which may introduce selection bias. Fourth, there was no inter-observer variability assessment despite experienced operators. Fifth, balloon-stretched diameter comparison was not performed, which is often considered a reference standard for sizing; device selection relied on 2D echocardiographic diameters. Finally, follow-up was limited to short-term outcomes (24 h), omitting long-term complications like device embolization or erosion.

## Conclusion

Based on our findings and the broader literature review, TTE can be a reliable and cost-effective first-line modality for pre-procedural assessment of ASD size, particularly for uncomplicated secundum ASDs in pediatric patients. TEE can be reserved for cases where TTE findings are inconclusive, for complex ASD morphologies, or when rim measurements are crucial for device selection. Implementing standardized protocols for both TTE and TEE measurements, including operator training and quality control measures, is crucial for enhancing the accuracy and consistency of ASD assessment across institutions. A collaborative approach involving cardiologists, echocardiographers, and other healthcare professionals, along with discussions with parents/guardians, is essential for determining the most appropriate assessment modality for each pediatric patient undergoing transcatheter ASD closure.

## Data Availability

The datasets generated and analyzed during the current study are available from the corresponding author upon reasonable request.

## Abbreviations

AR: Aortic rim
ASO: Amplatzer septal occluder
ASD: Atrial septal defect
AV: Atrio-ventricular
CHD: Congenital heart defect
ECG: Electrocardiogram
IVC: Inferior vena cava
ICU: Intermediate care unit
LAO: Left anterior oblique
LADEDT: Left atrial disc engagement–disengagement technique
LUPV: Left upper pulmonary vein
RUPV: Right upper pulmonary vein
SD: Standard deviation
SVC: Superior vena cava
TEE: Transesophageal echocardiography
TTE: Transthoracic echocardiography
2D: Two dimension

## References

[1] Zikarg YT, Yirdaw CT, Aragie TG. Prevalence of congenital septal defects among congenital heart defect patients in East Africa: A systematic review and meta-analysis. PLoS ONE. 2021;16(4):e0250006. 10.1371/journal.pone.0250006. 33886628 10.1371/journal.pone.0250006 PMC8062078

[2] Baruteau A-E, Petit J, Lambert V, Gouton M, Piot D, Brenot P, et al. Transcatheter Closure of Large Atrial Septal Defects. Circ Cardiovasc Interv. 2014;7(6):837–43. 10.1161/CIRCINTERVENTIONS.113.001254. 25423959 10.1161/CIRCINTERVENTIONS.113.001254

[3] Azhar AS. Safety and efficacy of transthoracic versus transesophageal echocardiography in transcatheter closure of atrial septal defects. Saudi Med J. 2016;37(11):1196–205. 27761557 10.15537/smj.2016.11.15617 PMC5303796

[4] Rana BS. Echocardiography guidance of atrial septal defect closure. J Thorac Dis. 2018;10(Suppl 24):S2899–908. 10.21037/jtd.2018.07.126. 30305950 10.21037/jtd.2018.07.126 PMC6174147

[5] Baruteau AE, Hascoët S, Fraisse A. Transthoracic echocardiography is a safe alternative for assessment and guidance of transcatheter closure of secundum atrial septal defect in children. J Thorac Dis. 2017;9(5):1247–56. 10.21037/jtd.2017.04.45. 28616275 10.21037/jtd.2017.04.47 PMC5465139

[6] Hascoet S, Hadeed K, Marchal P, Dulac Y, Alacoque X, Heitz F, et al. The relation between atrial septal defect shape, diameter, and area using three-dimensional transoesophageal echocardiography and balloon sizing during percutaneous closure in children. Eur Heart J Cardiovasc Imaging. 2015;16(7):747–55. 10.1093/ehjci/jeu316. 25617028 10.1093/ehjci/jeu316

[7] Khalaf AY. The promising role of different imaging diagnostic modalities for percutaneous transcatheter device closure of secundum atrial septal defect in pediatrics in Sohag University Hospital. Sohag Med J. 2023;27(2):87–94.

[8] Cinteza E, Vasile CM, Busnatu S, Armat I, Spinu AD, Vatasescu R et al. (2024) Can Artificial Intelligence Revolutionize the Diagnosis and Management of the Atrial Septal Defect in Children. Diagnostics 14. 10.3390/diagnostics14020132 PMC10814919 38248009

[9] Royse CF, Canty DJ, Faris J, Haji DL, Veltman M, Royse A. Core review: physician-performed ultrasound: the time has come for routine use in acute care medicine. Anesth Analg. 2012;115(5):1007–28. 23011559 10.1213/ANE.0b013e31826a79c1

[10] Xu WZ, Shou XY, Li JH, Yu JG, Zhang ZW, Yu J, et al. Non-fluoroscopic percutaneous transcatheter closure of atrial septal defects in children under transesophageal echocardiographic guidance. World J Pediatr. 2018;14(4):378–82. 10.1007/s12519-018-0179-x. 30141110 10.1007/s12519-018-0179-x PMC6154211

[11] Pan X, Li S, Hu S, Ouyang W, Zhang F, Zhang D, et al. Feasibility of transcatheter closure of atrial septal defect under the guidance of transthoracic echocardiography. Zhonghua xin xue guan bing za zhi. 2014;42(9):744–7. 25511094

[12] Zaidi A, Knight DS, Augustine DX, Harkness A, Oxborough D, Pearce K, et al. Echocardiographic Assessment of the Right Heart in Adults: A Practical Guideline from the British Society of Echocardiography. Echo Res Pract. 2020;7(1):G19–41. 10.1530/ERP-19-0051. 32105053 10.1530/ERP-19-0051 PMC7077526

[13] Zakaria J, Sébastien H, Céline G, François G, Lucia M, Claire D, et al. Long-Term Outcomes After Percutaneous Closure of Ostium Secundum Atrial Septal Defect in the Young. JACC Cardiovasc Interv. 2018;11(8):795–804. 29673513 10.1016/j.jcin.2018.01.262

[14] Özdemir E, Kırış T, Varış E, Emren SV, Nazlı C, Tokaç M. In-Hospital Cost of Transcatheter Closure Versus Surgical Closure of Secundum Atrial Septal Defect. Am J Cardiol. 2018;121(8):e31–2. 10.1016/j.amjcard.2018.03.094.

[15] Saritas T, Yucel IK, Demir IH, Demir F, Erdem A, Celebi A. Comparison of Transcatheter Atrial Septal Defect Closure in Children, Adolescents and adults: Differences, Challenges and Short-, Mid- and Long-Term Results. Korean Circ J. 2016;46(6):851–61. 27826346 10.4070/kcj.2016.46.6.851 PMC5099343

[16] Rossi RI, Cardoso CO, Machado PR, Francois LG, Horowitz ESK, Sarmento-Leite R. Transcatheter closure of atrial septal defect with Amplatzer device in children aged less than 10 years old: Immediate and late follow-up. Cathet Cardiovasc Intervent. 2008;71:231–6. 10.1002/ccd.21361 17985387

[17] Bartakian S, El-Said HG, Printz B, Moore JW. Prospective Randomized Trial of Transthoracic Echocardiography Versus Transesophageal Echocardiography for Assessment and Guidance of Transcatheter Closure of Atrial Septal Defects in Children Using the Amplatzer Septal Occluder. JACC Cardiovasc Interv. 2013;6(9):974–80. 10.1016/j.jcin.2013.05.007. 24050864 10.1016/j.jcin.2013.05.007

[18] Lang RM, Bierig M, Devereux RB, Flachskampf FA, Foster E, Pellikka PA, et al. Recommendations for chamber quantification. Eur J Echocardiogr. 2006;7(2):79–108. 10.1016/j.euje.2005.12.014. 16458610 10.1016/j.euje.2005.12.014

[19] Deng B, Chen K, Huang T, Wei Y, Liu Y, Yang L, et al. Assessment of atrial septal defect using 2D or real-time 3D transesophageal echocardiography and outcomes following transcatheter closure. Ann Transl Med. 2021;9(16):1309. 34532446 10.21037/atm-21-3206 PMC8422086

[20] Mesihović-Dinarević S, Begić Z, Halimić M, Kadić A, Gojak R. Reliability of transthoracic and transesophageal echocardiography in predicting the size of atrial septal defect. Acta Med Acad. 2012;41(2):145–53. 10.5644/ama2006-124.47. 23331389 10.5644/ama2006-124.47

[21] Johri AM, Witzke C, Solis J, Palacios IF, Inglessis I, Picard MH, et al. Real-Time Three-Dimensional Transesophageal Echocardiography in Patients with Secundum Atrial Septal Defects: Outcomes following Transcatheter Closure. J Am Soc Echocardiogr. 2011;24(4):431–7. 21262563 10.1016/j.echo.2010.12.011

[22] Pan XB, Ou-Yang WB, Pang KJ, Zhang FW, Wang SZ, Liu Y, et al. Percutaneous closure of atrial septal defects under transthoracic echocardiography guidance without fluoroscopy or intubation in children. J Interv Cardiol. 2015;28:390–5. 26077469 10.1111/joic.12214

[23] Erdem A, Sarıtas T, Zeybek C, Yucel IK, Erol N, Demir H, et al. Transthoracic echocardiographic guidance during transcatheter closure of atrial septal defects in children and adults. Int J Cardiovasc Imaging. 2013;29:53–61. 21833775 10.1007/s10554-011-9933-z

[24] Lan Q, Wu F, Ye X, Wang S, Zhong J. Intracardiac vs. transesophageal echocardiography for guiding transcatheter closure of interatrial communications: a systematic review and meta-analysis. Front Cardiovasc Med. 2023;10:1082663. 10.3389/fcvm.2023.1082663. 37215547 10.3389/fcvm.2023.1082663 PMC10198467

[25] Magni G, Hijazi ZM, Pandian NG, Delabays A, Sugeng L, Laskari C, et al. Two- and Three-Dimensional Transesophageal Echocardiography in Patient Selection and Assessment of Atrial Septal Defect Closure by the New DAS–Angel Wings Device. Circulation. 1997;96(6):1722–8. 10.1161/01.CIR.96.6.1722. 9323052 10.1161/01.cir.96.6.1722

[26] Vesal A, Moradian M, Ghasemnezhad M, Tabib A, Rashidi F, Disney PJ et al. (2022) Comparison Between 2D Transthoracic Echocardiography, Transesophageal Echocardiography, and Balloon Sizing Methods for Device Size Selection in Pediatric Patients Undergoing Transcatheter Closure of Atrial Septal Defects. Iran Heart J 23(1).

